# NT-proBNP <95 ng/l can exclude pulmonary hypertension on echocardiography at diagnostic workup in patients with interstitial lung disease

**DOI:** 10.3402/ecrj.v3.32027

**Published:** 2016-07-29

**Authors:** Charlotte Andersen, Søren Mellemkjær, Ole Hilberg, Elisabeth Bendstrup

**Affiliations:** 1 Department of Clinical Pharmacology, Aarhus University Hospital, Aarhus, Denmark; 2 Department of Cardiology, Aarhus University Hospital, Aarhus, Denmark; 3 Department of Respiratory Diseases and Allergy, Aarhus University Hospital, Aarhus, Denmark

**Keywords:** lung fibrosis, biomarker, brain natriuretic peptides, diagnosis, cor pulmonale, idiopathic pulmonary fibrosis, IPF

## Abstract

**Background:**

Pulmonary hypertension (PH) is a serious complication to interstitial lung disease (ILD) and has a poor prognosis. PH is often diagnosed by screening with echocardiography followed by right heart catheterisation. A previous study has shown that a value of NT-pro-brain natriuretic peptide (NT-proBNP) <95 ng/l could be used to rule out PH in patients with ILD.

**Aim:**

To evaluate this rule-out test for PH in a new cohort of incident patients with ILD.

**Methods:**

An established database with data from 148 consecutive patients referred from January 2012 to October 2014 was used to identify patients and obtain data from echocardiography, NT-proBNP, diagnosis and lung function. Signs of PH on echocardiography were defined as a tricuspid pressure gradient (TR) ≥40 mmHg, decreased right ventricular systolic function or dilatation. Sensitivity, specificity, negative predictive value (NPV) and positive predictive value (PPV) of NT-proBNP >95 ng/l for signs of PH on echocardiography were calculated. The study was approved by the Danish Health Authority.

**Results:**

In 118 patients, data from both echocardiography and measurements of NT-proBNP were available. Eleven of these were screened positive for PH on echocardiography. Sensitivity, specificity, NPV and PPV of NT-proBNP <95 ng/l for PH were 100, 44, 16 and 100%, respectively. Furthermore, no patients with left heart failure as the cause of dyspnoea were missed using this cut-off value.

**Conclusion:**

NT-proBNP <95 ng/l precludes a positive echocardiographic screen for PH in ILD patients at referral for diagnostic workup.

Pulmonary hypertension (PH) is a serious complication to interstitial lung disease (ILD), which worsens prognosis and impairs exercise capacity (1). The gold standard for diagnosis is right heart catheterisation, which is an invasive method, but echocardiography is often used as a screening tool and has a good sensitivity for PH (2). However, echocardiography may not be immediately available for pulmonologists, and therefore, biomarkers for PH are desirable. Several biomarkers have been proposed, but so far the brain natriuretic peptides (BNP) and NT-proBNP secreted from the heart are the only established biomarkers for PH in clinical use (3, 4). In a previous cross-sectional study with prevalent and incident patients with ILD, we found that a value of NT-proBNP <95 ng/l was able to preclude the presence of PH (5). The purpose of this study was to evaluate this result in a new cohort of incident patients with ILD at the time of referral to our department.

## Methods

### Database

From January 2012, all patients referred to the Department of Respiratory Diseases and Allergy, Aarhus University Hospital, for the evaluation of pulmonary fibrosis were registered in the Danish National ILD database. In October 2015, the data from 148 patients referred from January 2012 to October 2014 had been entered. Demographic parameters, ILD-diagnosis, lung function parameters, the 6-minute walk test (6MWT), diagnoses of cardiac co-morbidities or diabetes, echocardiography and levels of NT-proBNP were noted at baseline. The study was approved by the Danish Health Authority.

### Diagnosis

Diagnoses of ILDs were obtained after standard workup with clinical examination, lung function tests, high-resolution computed tomography scan, bronchoscopy with bronchoalveolar lavage and either trancbronchial- or video-assisted thoracoscopic lung biopsy, if indicated.

### Echocardiography

At the time of referral to our department, patients are routinely given echocardiography performed by a cardiologist, if this has not recently been performed. The purpose is to screen for PH and other structural or functional heart disease. Data for this study was obtained from the description of the echocardiography in the patient record. A positive screen for PH was defined as a tricuspid regurgitation pressure gradient (TR) of 40 mmHg or more, decreased tricuspid annular plane systolic excursion (TAPSE) <1.8 cm and/or dilatation of the right ventricle. In cases where TR and TAPSE were not mentioned in the echocardiography description, the echocardiograms were inspected by the authors if possible, and the overall conclusion on whether PH was present or not was used to classify the patient as screened positive or negative. In some cases, TAPSE was reported <1.8 cm, while the overall conclusion was that PH was not suspected. In these cases, the overall conclusion was used for final classification. It was also noted if valvular abnormalities, or left ventricular systolic or diastolic function were observed.

### NT-proBNP

The concentration of NT-proBNP in venous blood samples were analysed at the hospital's CME-accredited laboratory at the Department of Clinical Biochemistry using a standard procedure (cobas 6000 E/C, Roche, West Sussex, England). Normal range <300 ng/l.

### Lung function

Spirometry, body plethysmography and a determination of diffusion capacity for carbon monoxide (DL_CO_) (Zan 500 Body, nSpire Health Inc, Louisville, Colorado, USA; PFT pro Body, Jaeger, Hoeckberg, Germany; Spirometer model 6800, Vitalograph, Ennis, Ireland) were undertaken in accordance with the American Thoracic Society guidelines (6). Predicted values were calculated as recommended by the European Respiratory Society (7).

### The 6-minute walk test

The 6MWT was performed following guidelines from the ATS (8). The tests were conducted on a 30-m track. Patients on long-term oxygen used their prescribed supplemental oxygen dosage during the test.

### Data analysis

Data were analysed in Stata/IC 10 (StataCorp, College Station, Texas USA). The discriminatory power for PH on echocardiography for NT-proBNP was analysed by receiver operating characteristic (ROC) curves. Sensitivity, specificity, positive predictive value (PPV) and negative predictive value (NPV) were calculated for the pre-defined cut-off value of 95 ng/l.

The normal distribution of data was checked by histograms and q-norm plots. Parametric data were analysed using Student's *t*-test, non-parametric data by rank sum test. Parametric data are expressed as mean±standard error of the mean, non-parametric as medians with interquartile ranges. Sensitivity, specificity, PPV and NPV are expressed with 95% CIs. A *p*-value <0.05 was regarded as statistically significant.

## Results

### Patients included in the final analysis

**Fig. 1:**
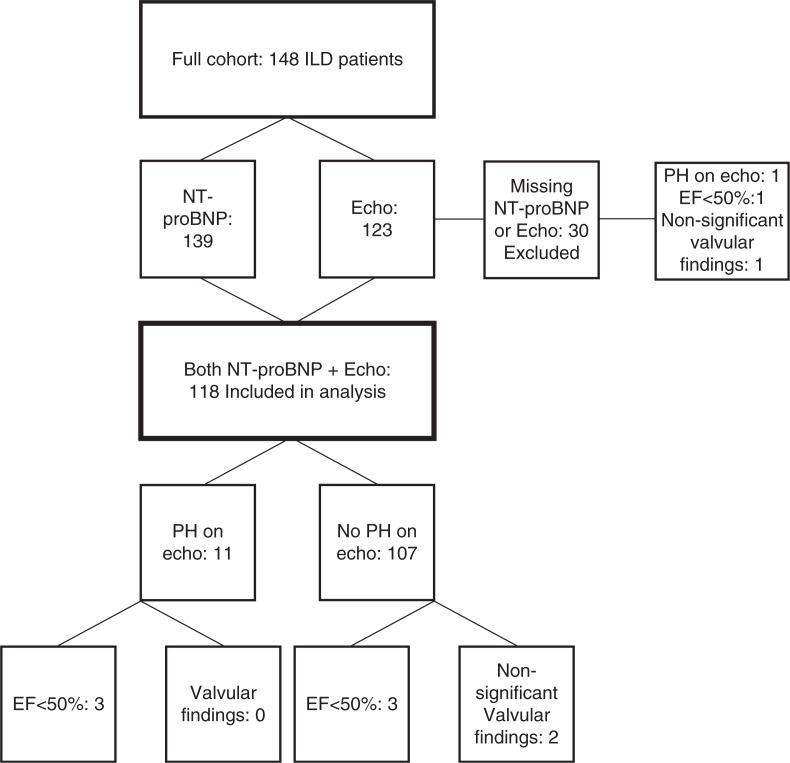
Flow chart showing the number of patients included in final analysis and the results of echocardiography in the respective subgroups, ILD, interstitial lung disease; Echo, echocardiography; PH, pulmonary hypertension; EF, left ventricular ejection fraction.

In 139 patients, NT-proBNP were measured, and echocardiography was performed in 123 patients. In 118 patients, data from both NT-proBNP and echocardiography were available, and these 118 patients were included in the final analysis (Fig. 1).

**Fig. 2:**
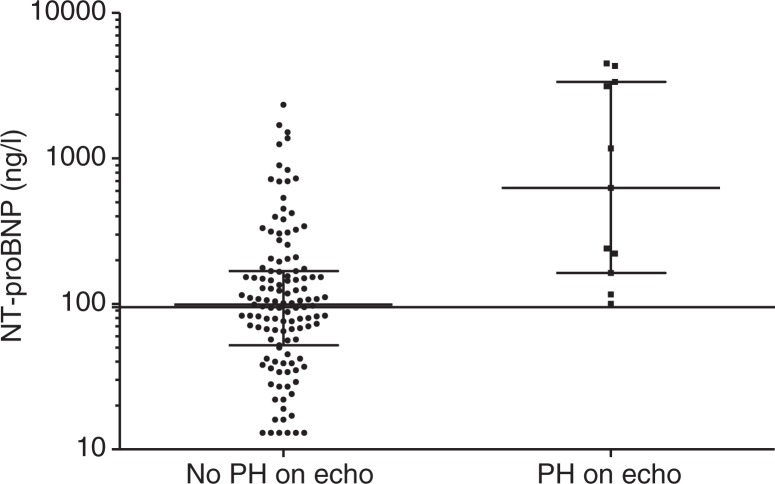
The values of NT-proBNP in patients with and without PH (pulmonary hypertension) on echocardiography. The vertical line at 95 ng/l. Error bars represent the median and interquartile range. *p*=0.0003.

### Demographics

The distribution of diagnoses and prevalence of cardiovascular co-morbidities of whole cohort and the 118 patients included in the final analysis are shown in Table 1.

**Table 1:** Diagnoses and comorbidity in the full cohort and the 118 patients included in the final analysis

	Full cohort (*n*=148)	Patients in analysis (*n*=118)
Diagnosis *n* (percent)		
IPF	48 (32)	43 (36)
NSIP	8 (5)	8 (7)
DIP	4 (3)	3 (3)
Hypersensitivity pneumonitis	5 (3)	4 (3)
ILD related to collagen vascular disease	13 (9)	11 (9)
Other ILD	40 (27)	25 (21)
Unclassifiable ILD	30 (20)	24 (20)
Co-morbidity		
Chronic left heart failure^a^	10 (7)	9 (8)
Ischemic heart disease	13 (9)	13 (10)
Atrial fibrillation	13 (9)	11 (9)
Diabetes	4 (3)	3 (3)

IPF, idiopathic pulmonary fibrosis; NSIP, non-specific interstitial pneumonia; DIP, desquamative interstitial pneumonia; ILD, interstitial lung disease.

a Some of these were well treated and had normal left ventricular ejection fractions.

### Echocardiography to define patients with PH

Echocardiography was performed on 123 out of the total 148 patients. Measurements of TR and TAPSE were obtained for 74 and 82 patients, respectively.

It was concluded that 12 patients had signs of PH on echocardiography, based on a TR ≥40 mmHg (*n*=11) (mean TR 48±2.9 mmHg) or TAPSE = 1.3 cm and dilatation of the right ventricle (*n*=1). An additional eight patients had a TAPSE <1.8 cm, but with normal dimensions of the right ventricle and absent or low TR. These were considered screened negative for PH. Seven patients had a left ventricular ejection fraction (EF) <50% and three patients had mild and non-significant valvular abnormalities (mild aortic valve sclerosis and mild aortic insufficiency (*n*=1), mechanical aortic valve (*n*=1) and mild aortic valve sclerosis (*n*=1). None of these three had impaired left ventricular systolic function.

### NT-proBNP as a diagnostic tool

Analysis of NT-proBNP at referral was performed for 139 patients. For 118 patients, both data from echocardiography and NT-proBNP were obtained. Eleven of these had PH on echocardiography. In 53 patients, echocardiography was performed within 3 days of referral and NT-proBNP measurements. Median time between echocardiography and NT-proBNP measurement was 0.5 (0–4) months. Log transformed NT-proBNP values were normally distributed.

NT-proBNP was significantly higher in patients screened positive for PH by echocardiography (*p*=0.0003). In 44% of the 139 with measurements of NT-proBNP and 41% of the 118 patients included in the final analysis, NT-proBNP was <95 ng/l (Fig. 1).

The area under the ROC curve for NT-proBNP to detect PH on echocardiography was 0.83 (95% CI 0.71–0.95). Sensitivity, specificity, NPV and PPV of NT-proBNP <95 ng/l for PH were 100 (72–100)%, 44 (34–54)%, 16 (8–26)% and 100 (93–100)%, respectively.

Of the six patients included in the analysis with EF <50% (Fig. 1), none had NT-proBNP <95 ng/l (range 240–4,493 ng/l). Of the two patients with non-significant valvular findings included in the analysis, one patient who had mild aortic valve sclerosis and insufficiency and an EF of 60% had NT-proBNP <95 ng/l (40 ng/l).

### Lung function parameters and 6MWT

Lung function parameters and 6MWT distance for patients in different categories are shown in Table 2. The 118 patients included in the analysis were older, had lower DLCo, and a shorter 6MWT than the 30 patients not included in the analysis. Compared to those who did not have echocardiography performed, the 123 patients, in whom echocardiography was performed, were older, had a lower diffusion capacity and a shorter 6MWT distance. Six minute walk test was significantly lower in patients screened positive for PH compared to those screened negative.

**Table 2:** Demographics, lung function parameters, 6-min walk test and NT-proBNP

	Total (*n*=148)	Included in analysis (*n*=118)	Excluded from analysis (*n*=30)	Echo performed (*n*=123)	No echo (*n*=25)	No PH on echo (*n*=111)	PH on echo (*n*=12)
Age (years)	64.5	66 (64–69)	55 (48–62)^a^	66 (64–68)	54 (46–62)^b^	66 (64–69)	69 (63–76)
Female (percent)	39	40	30	32	40	38	58
TLC (percentage of expected)	78 (75–82)	78 (75–81)	80 (72–89)	77 (74–81)	84 (76–94)	78 (74–81)	73 (65–81)
DL_CO_ (percentage of expected)	51 (48–55)	49 (45–52)	61 (50–70)^a^	48 (45–51)	66 (56–77)^b^	49 (46–52)	40 (27–53)
FEV1 (percentage of expected)	80 (76–84)	80 (77–84)	84 (75–93)	79 (76–83)	85 (76–95)	78 (76–84)	75 (59–91)
FVC (percentage of expected)	92 (77–107)	88 (74–101)	84 (74–94)	87 (74–100)	87 (76–98)	88 (74–102)	78 (63–93)
6MWT distance (meters)	433 (410–455)	420 (396–444)	488 (431–543)^a^	416 (392–440)	518 (463–573)^b^	424 (399–448)	327 (237–417)^c^

Echo, echocardiography; TLC, total lung capacity; DL_CO_, diffusion capacity; FEV1, forced expiratory capacity in 1 sec; FVC, forced vital capacity; 6MWT, 6-minute walk test.

Data are expressed as means with 95% confidence intervals, unless otherwise stated.

a *p*<0.05 versus included in analysis.

b *p*<0.05 versus echo performed.

c *p*<0.05 versus no PH on echo.

## Discussion

The main finding of this study was that a value of NT-proBNP <95 ng/l could be confirmed as a rule-out test for PH in ILD patients as indicated in a former study (5). Furthermore, no cases of left ventricular dysfunction were missed as the cause for dyspnoea in the patients at referral.

### NT-proBNP as a biomarker

BNPs are secreted in response to cardiac strain (9), and since pathological changes in the pulmonary arteries occur prior to cardiac affection in PH, pulmonary derived biomarkers would be optimal to detect early PH. However, although such biomarkers are hoped to emerge and promising results have recently been obtained with VEGF-family members (10), they are not yet established in clinical use.

Analysis of NT-proBNP is available in several laboratories, and the normal upper limit is 300 ng/l at our laboratory. While patients with PH can have normal values of BNPs (10), other studies are in agreement with our finding that a value in the lower spectrum of normal can be useful to discriminate pulmonary from cardiac causes of dyspnoea. In one study, levels of 93 ng/l and 144 ng/l for men and women referred with dyspnoea, respectively, had a sensitivity of 90% for detecting cardiac abnormalities (11). Most of the patients in that study had COPD or asthma. Another study found that a value of NT-proBNP <80 pg/ml was able to exclude the presence of PH in patients screened positive for PH by echocardiography with a systolic right ventricular pressure (SPAP) >36 mmHg at workup for suspected pulmonary arterial hypertension (PAH) (12). However, due to demographic differences such as age and co-morbidities between the study subjects in prior studies and incident patients with ILD, it is critical to investigate the latter population in particular.

Not all patients had echocardiography and NT-proBNP measurements performed simultaneously in this study. A recent study from patients with mild to moderate restrictive ILD showed that the pulmonary pressure measured by right heart catheterisation was quite stable over a period of 48 weeks (13). Hence, the significance of the median interval of 0.5 months or even up to 4 months between the measurements is probably limited.

### Echocardiographic screening

Right heart catheterisation is the only means to establish a diagnosis of PH with certainty. However, a study by Arcasoy, et al. showed that echocardiographic measurements of the SPAP, with a cut-off value of 45 mmHg, and an evaluation of right ventricular abnormalities, had a fair sensitivity for PH in patients with chronic lung disease (2). In this study, we rely mainly on a TR >40 mmHg as an indicator for PH. Although it is documented that TAPSE <1.8 cm has prognostic value in patients with PAH (14), the diagnostic use of an isolated TAPSE value is not validated in patients with lung disease. Reflecting daily practice, TAPSE was included in the overall evaluation of the right ventricle in this study. The risk of missing patients with PH by echocardiography is regarded as acceptable for screening patients with suspected ILD at referral at our department, although it cannot be fully eliminated.

### Study limitations

The main limitation of the study is the limited size, with only 11 cases of patients screened positive for PH. On the other hand, we find that the validation of the former result in a new independent cohort is valuable.

Furthermore, differences in age, diffusion capacity and 6MWT were observed between patients for whom echocardiography had been omitted, compared to those for whom it was performed. There was also a trend toward lower values of NT-proBNP in those in which echocardiography was omitted. This could mean that echocardiography may have been omitted in patients with low NT-proBNP, which is a source of bias. In most cases, however, echocardiography was already booked prior to the measurement of NT-proBNP, as this was standard in the diagnostic workup routine at our department. We do not know whether the cardiologists were aware of the NT-proBNP value before performing the echocardiography in each case. Because NT-proBNP is not yet part of any formal algorithm for the diagnosis of PH in ILD patients, it should not have influenced conclusions regarding the presence of PH. Hence, we speculate that any related bias is limited.

## Conclusion

The formerly proposed value of NT-proBNP <95 ng/l as a rule-out test for PH in patients with ILD was confirmed in a new cohort of ILD patients. This suggests that routine echocardiographic screening for PH may be omitted in patients with an NT-proBNP value <95 ng/l at referral for workup of suspected ILD.
